# A profile of children’s physical activity data from the 2012 and 2015 health survey for England

**DOI:** 10.1186/s12889-022-14150-4

**Published:** 2022-09-20

**Authors:** Jamie Sims, Karen Milton, Charlie Foster, Peter Scarborough

**Affiliations:** 1 Department of Population Health, University of Oxford, Nuffield, Old Road Campus, OX3 7LF Oxford, UK; 2 Department of Sport, Health Sciences and Social Work, Oxford Brooks University, Headington Campus, OX3 0BP Oxford, UK; 3 Norwich Medical School, University of East Anglia, Norwich Research Park, Norwich, NR4 7TJ UK; 4 School for Policy Studies, Social Science Complex, 8 Priory Road, Bristol, BS8 1TZ UK

**Keywords:** Physical activity, Health survey for England, Child health, Ethnicity

## Abstract

**Background:**

Low childhood physical activity levels constitute an important modifiable risk for adult non-communicable disease incidence and subsequent socio-economic burden, but few publications have explored age and sex related patterns within the UK population. The aims were to profile child physical activity data from the Health Survey for England from 2012 (1,732 respondents) and 2015 (5,346 respondents).

**Methods:**

Reported physical activity episodes were converted to metabolic equivalents with reference to child-specific compendiums. Physical activity levels were aggregated for each domain, and again to produce total physical activity estimates. Contributions from each domain to total physical activity were explored, stratifying for age, sex, socio-economic deprivation, ethnicity, and weight status. Further analyses were run stratifying for physical activity levels. Few differences were detected between the survey iterations.

**Results:**

Boys reported higher absolute levels of physical activity at all ages and across all domains. For boys and girls, informal activity reduces with age. For boys this reduction is largely mitigated by increased formal sport, but this is not the case for girls. Absolute levels of school activity and active travel remained consistent regardless of total physical activity, thereby comprising an increasingly important proportion of total physical activity for less active children.

**Conclusions:**

We recommend a specific focus on establishing and maintaining girl’s participation in formal sport thorough their teenage years, and a recognition and consolidation of the important role played by active travel and school-based physical activity for the least active children.

## Background

Physical activity in children has been hypothesised as being important in reducing the risk of non-communicable diseases across the life-span [1]. Physical inactivity tends to cluster with overweight and obesity [2], which itself possesses independent associations with key risk factors of poor health [3]. Physical inactivity and obesity have been shown to track more strongly than physical activity into adulthood [4, 5], where they constitute independent risk factors for type 2 diabetes [6], cardiovascular disease [7], and cancer [8] incidence and mortality. These factors combine with indirect consequences that also arise from childhood inactivity and obesity, including psychological, social, and economic issues, to compound negative health impacts such as child self-esteem [9]. The financial burden associated with the healthcare costs to service the population is substantial [10], constituting a clear motivation for governments to promote cost-effective interventions to increase childhood physical activity as part of policy for reducing non-communicable disease morbidity and mortality in later life [5, 11].

While the case for intervention to increase childhood physical activity levels is clear, decisions must be supported by adequate data on population trends, facilitating the identification of priority areas for intervention and programming opportunities for children to participate in physical activity. Since 2005, Physical Activity Report Cards [12] have been completed regularly for participating nations, reporting on and ‘grading’ the provision of physical activity opportunities for children. A key element of this report is the quality of surveillance and evidence from which decision-makers may draw relevant information, and on which basis which England achieved a C- in the 2018 report [13].

Within England, the Health Survey for England (HSE) is used to inform intervention and policy, as it is essential to have access to regularly updated data on the population-level patterns of physical activity. A specific module on physical activity has been periodically included within the survey, including 2004, 2008 and 2012. Respondents include both adults and children within households in England, with children’s responses either completed or verified by a parent. In addition, physical activity was assessed in a larger ‘boost’ sample of children in 2015.

An analysis of the subset of children meeting physical activity guidelines from the 2008 survey was completed by Payne, Townsend [14], concluding that active play was the largest contributor to total childhood physical activity for both sexes, although this decreased with age to be partially replaced by formal sport participation. Walking was the second largest contributor for both sexes, but the overall contribution from active travel was minimal. Formal sport was more popular among boys than girls at all age-groups but reduced in popularity as socioeconomic status declined. However, this analysis was restricted to ‘active’ children and did not include school-based physical activity or physical education, and therefore provides limited insight into how to target interventions to address inactivity. There remains a requirement for a comprehensive analysis of childhood physical activity patterns to inform future interventions or policy given the need to specifically target those children not achieving healthy levels of physical activity.

While a domain-level analysis of physical activity patterns within the English population provides broad insights, the HSE permits analysis of additional demographic variables hypothesised to moderate physical activity behaviour. In particular, levels of physical activity have been shown or suggested to vary by sex (e.g. [15]), age (e.g. [16]), and adiposity (e.g. [17]), as well as ethnicity and socio-economic deprivation (e.g. [18, 19]), and a more nuanced analysis is therefore possible in order to inform targeted intervention for those most in need of support.

The aim of this paper is to identify the contribution from specific activity-domains to total physical activity levels in children aged from two to fifteen years, including analyses of differences across key demographic variables. We investigate variations in physical activity patterns by age and sex across the full distribution of child physical activity levels and identify differences in patterns between 2012 and 2015 survey iterations. We also investigate domain-specific contribution to total physical activity levels by level of activity, extending the work of Payne, Townsend [14] to incorporate the full spectrum of child activity.

## Methods

### Sources of data

The HSE is administered via household interview. The HSE uses a multi-stage stratified probability approach to provide annual, nationally representative data for a cross-section of the population of England, conducted throughout the year to control for seasonality. The HSE uses the Physical Activity and Sedentary Behaviour Assessment Questionnaire (PASBAQ) to collect self-reported physical activity behaviour over the seven days immediately preceding the interview [20]. Children aged above twelve years were interviewed directly, while for younger children the questionnaire was completed with both a parent and the target child present. Data on a maximum of two children per household were collected. The PASBAQ collects the duration and frequency of participation in specific forms of physical activity across walking and cycling, domestic activity, formal sport, and informal activity domains, but does not include school curriculum time physical activity. A broad estimation of intensity is also gathered for each episode of physical activity participation [20, 21]. Episodes of participation in specific periods of at least ten minutes or more are recorded and summed to provide domain-specific totals which are then summed to produce a grand total for minutes of physical activity per week. In the HSE 2015 iteration, a single item requesting an estimate of school-based physical activity in minutes over the past week was included. This study used data from the HSE 2012 and 2015, the two most recent survey iterations to include the physical activity module. The raw data for both HSE iterations is made available for download by UCL Data Unit/NHS Digital and as a result no consent or ethics procedures were required for this study.

### Treatment of data

The raw data were downloaded for all respondents from the HSE 2012 (*n* = 10,333) and 2015 (*n* = 13,748) into the Stata 15 statistics package [22]. Demographic and social data were retained for analysis, including age, sex, identified ethnic group, index of multiple deprivation, rurality of residence, and BMI status. Index of multiple deprivation is a composite score of the locality based on weighted estimates of: income deprivation; employment deprivation; education, skills and training deprivation; health deprivation and disability; crime; barriers to housing and services; and living environment deprivation [23]. Scores are typically reported as quintiles within each survey, meaning that scores cannot be compared directly between survey years as quintile bands vary.

We excluded all respondents aged above fifteen (8,290 from HSE 2012 & 8,034 from HSE 2015) along with those under two years of age (311 from HSE 2012 & 278 from HSE 2015). Both surveys incorporated weighting to address sampling bias due to oversampling of households from sparsely populated areas, dwelling and household unit selection, and non-response calibration. The HSE 2015 also included a weighting specifically for child respondents to address potential under-selection from households with more than two children [24].

For each recorded type of physical activity across all domains, metabolic equivalence (METs) estimates were referenced from physical activity equivalence compendiums [25–27]. Estimates of MET minutes per week were generated by multiplying the number of minutes per week by the associated MET estimate for each specific activity. Relevant activity estimates were then summed to provide an estimate of MET minutes per week for each domain of physical activity: active travel (walking or cycling to school); formal physical activity (structured extra-curricular sport and exercise); informal physical activity (unstructured activities such as play or skipping); non-specified physical activity (other forms of physical activity not covered by pre-defined domain-specific questions); and for the HSE 2015 only, school physical activity (all activity occurring within the school curriculum including break and lunch times). These domain-specific estimates were then summed to provide a total amount of weekly exertion through physical activity.

### Statistical analysis

For both survey years, total minutes of reported physical activity and mean contributions of physical activity domains towards total activity were calculated by age and sex. Descriptive investigation was conducted for total and domain-specific mean physical activity, stratified by sex, demographics, and social factors. A series of one-way ANOVAs were conducted on total and domain-specific estimates, controlling for age and sex on subsequent variables. For each survey year, total weekly physical exertion was divided into quintiles, providing stratification by physical activity level to ascertain whether physical activity differs by type as well as by overall quantity at progressive levels of total activity. A series of t-tests were run to explore differences between 2012 and 2015 data. Survey weighted estimates were used throughout these analyses.

## Results

For the HSE 2012, a sample of 1,732 unique children were included in the present analysis, of which 50% were female. Table 1 presents means of total and domain-specific MET mins per week by individual factors for the HSE 2012. For the HSE 2015, 5,346 unique individuals were entered into the analysis, of which 49% were female. Table 2 presents means of total and domain-specific MET mins per week by individual factors for the HSE 2015. The data are presented in separate tables as, while the means and standard deviations broadly correspond across datasets, the inclusion of school physical activity within HSE 2015 renders the estimates not directly comparable. Comparisons between HSE 2012 and 2015 data reveal that although occasional differences are observed between isolated subgroups, no systematic differences occur between survey iterations.

**Table 1:** 2012 total and domain-specific MET minutes/week, and percentage contributions, by sex, age, weight status

Category	***n***	**Total**	**Active Travel**		**Non-Specific**		**Formal**		**Informal**	
		**Mean (SD)**	**Mean (SD)**	**%**	**Mean (SD)**	**%**	**Mean (SD)**	**%**	**Mean (SD)**	**%**
Cohort	1732	3326.3 (3054.4)	183.2 (275.4)	5.5	112.5 (494.3)	3.4	470.4 (934.7)	14.1	2560.3 (2840.0)	79.7
Sex										
Male	862	3682.6 (3297.6)	189.0 (288.5)	5.4	106.1 (430.0)	3.1	611.6 (1086.9)	18.8	2775.8 (2571.9)	72.7
Female	870	2973.4 (2749.5)	177.5 (261.8)	6.0	118.8 (550.9)	4.0	330.4 (728.4)	11.9	2346.8 (2173.8)	78.1
Effect by Sex		t = 4.86 **	t = 0.87		t = -0.53		t = 6.33 **		t = 3.15 **	
Age Group										
2–4	428	3636.0 (3725.2)	93.2 (160.7)	2.6	16.0 (97.8)	0.4	200.6 (616.4)	5.5	3326.2 (3643.4)	91.5
5–7	376	3139.2 (2511.6)	160.7 (183.1)	5.1	78.0 (292.8)	2.5	372.3 (586.0)	11.9	2528.3 (2497.8)	80.5
8–10	368	3355.8 (2717.5)	169.4 (210.0)	5.0	136.5 (387.4)	4.1	606.0 (1009.2)	18.1	2444.0 (2481.2)	72.8
11–12	260	3561.9 (3205.3)	289.2 (385.5)	8.1	206.1 (815.0)	5.8	710.7 (1190.9)	20.0	2355.9 (2676.7)	66.1
13–15	300	2878.6 (3054.4)	264.9 (385.1)	9.2	182.8 (711.2)	6.4	603.5 (1183.4)	21.0	1827.4 (2308.6)	63.5
Effect by Age		*F* = 4.42 **	*F* = 28.58 **		*F* = 8.23 **		*F* = 18.34 **		*F* = 14.92 **	
Weight Status										
Normal	947	3254.1 (2961.2)	186.7 (274.0)	5.7	127.5 (503.7)	3.9	500.9 (963.2)	15.4	2439.0 (2742.6)	75.0
Overweight	190	3566.9 (3345.6)	213.7 (311.7)	6.0	174.0 (812.4)	4.9	534.6 (1080.9)	15.0	2644.5 (3017.5)	74.1
Obese	184	3452.3 (3074.7)	195.4 (287.0)	5.7	64.7 (215.3)	1.9	603.9 (1130.1)	17.5	2588.3 (2667.2)	75.0
Effect by Weight Status		*F* = 0.85	*F* = 0.53		*F* = 3.55 *		*F* = 0.18		*F* = 0.87	

^*^p < 0.05

^**^p < 0.01

**Table 2:** 2015 total and domain-specific MET minutes/week, and percentage contributions, by sex, age, and weight status

Category	**n**	**Total**	**Active Travel**		**Non-Specific**		**Formal**		**Informal**		**School**	
		**Mean (SD)**	**Mean (SD)**	**%**	**Mean (SD)**	**%**	**Mean (SD)**	**%**	**Mean (SD)**	**%**	**Mean (SD)**	**%**
Cohort	5436	3792.0 (3418.8)	160.0 (263.5)	4.2	75.5 (344.2)	2.0	535.4 (1220.3)	14.1	2795.6 (3096.6)	73.7	225.7 (337.1)	6.0
Sex												
Male	2698	4131.1 (3616.3)	156.7† (255.3)	3.8	74.9† (358.3)	1.8	697.9 (1360.9)	16.9	2970.7 (3208.7)	71.9	230.8 (343.4)	5.6
Female	2738	3457.8 (3338.8)	162.8 (271.5)	4.7	76.1† (329.8)	2.2	375.2 (1039.5)	10.9	2623.1 (2511.7)	75.9	220.7 (330.8)	6.4
Effect by Sex		t = 7.29 **	t = -0.85		t = -0.13		t = 9.84 **		t = 4.15 **		t = 1.10	
Age Group												
2–4	1281	3926.5 (3785.1)	88.7 (154.6)	2.3	26.2 (175.5)	0.7	206.4 (552.9)	5.3	3605.1 (3712.9)	91.8	22.4 (135.6)	0.6
5–7	1265	3531.1 (3119.3)	125.0† (184.4)	3.5	56.1 (231.0)	1.6	421.9 (1034.4)	11.9	2927.8† (2875.0)	82.9	164.7 (239.7)	4.7
8–10	1171	3848.8 (3591.3)	125.3† (186.8)	3.3	103.5 (361.7)	2.7	690.2 (1478.9)	17.9	2929.7† (3091.7)	76.1	197.4 (282.2)	5.1
11–12	730	3470.4 (3166.9)	252.3 (353.9)	7.2	102.0† (393.2)	2.9	799.2 (1491.9)	23.0	2316.9 (2596.8)	66.8	274.4 (392.8)	7.9
13–15	989	2880.8 (3124.7)	268.7 (382.3)	9.3	111.4 (517.8)	3.9	728.3 (1386.1)	25.3	1772.4 (2430.9)	61.5	266.3 (415.9)	9.2
Effect by Age		*F *= 16.01 **	*F *= 104.44 **		*F *= 15.22 **		*F﻿ *= 53.04 **		*F *= 55.36 **		*F *= 206.47 **	
Weight Status												
Normal	3148	3665.2† (3447.8)	157.9† (260.9)	4.3	86.8† (378.7)	2.4	563.8 (1300.8)	15.4	2856.6 (3099.0)	77.9	182.2 (314.8)	5.0
Overweight	644	3505.2 (3192.7)	167.7† (260.0)	4.8	68.6† (290.8)	2.0	577.7 (1031.1)	16.5	2691.2† (3025.7)	76.8	177.5 (296.6)	5.1
Obese	639	3219.9 (3188.8)	184.7 (271.0)	5.7	77.8 (378.2)	2.4	563.7 (1269.7)	17.5	2393.7 (2713.2)	74.3	210.3 (374.6)	6.5
Effect by Weight Status		*F* = 6.04 **	*F* = 0.85		*F* = 0.61		*F* = 2.53		*F* = 4.82 **		*F* = 0.55	

^*^p < 0.05

^**^p < 0.01, † Significant difference (*p* < 0.05) between 2012 and 2015 data

### Effect by sex

For the HSE 2012, boys reported higher total levels of physical activity than girls (*t*_1732_ = 4.86, *p* < 0.001). There were only minor differences between boys and girls on totals for active travel and non-specific physical activity, with no specific differences emerging when stratified by age group. Boys accrued a higher percentage of physical activity from formal sports than girls (*t*_1674_ = 5.47, *p* < 0.001), while girls conversely recruited a higher percentage of physical activity from informal activities than boys (*t*_1674_ = -2.83, p = 0.005), although boys still reported higher absolute levels of informal physical activity (*t*_1730_ = 3.15, *p* = 0.005). When stratified by age group, informal activity only differed significantly between sexes within the 13- to 15-year-old group, but formal activity showed large differences between sexes in all school-age groups, with boys consistently achieving more informal activity and total physical activity than girls.

A similar pattern was observed within the HSE 2015 data, with boys reporting significantly higher physical activity levels than girls on total MET mins per week including (*t*_5436_ = 7.29, *p* < 0.001) and excluding (*t*_5436_ = 7.19, *p* < 0.001) school physical activity. These differences between boys and girls largely persisted when stratified by age. Boys again recruited a higher percentage of total physical activity than girls from formal activity (*t*_5122_ = 10.30, *p* < 0.001). The remaining domains of active travel, non-specific, informal, and school physical activity showed little variation by sex.

### Effect by age

**Fig. 1:**
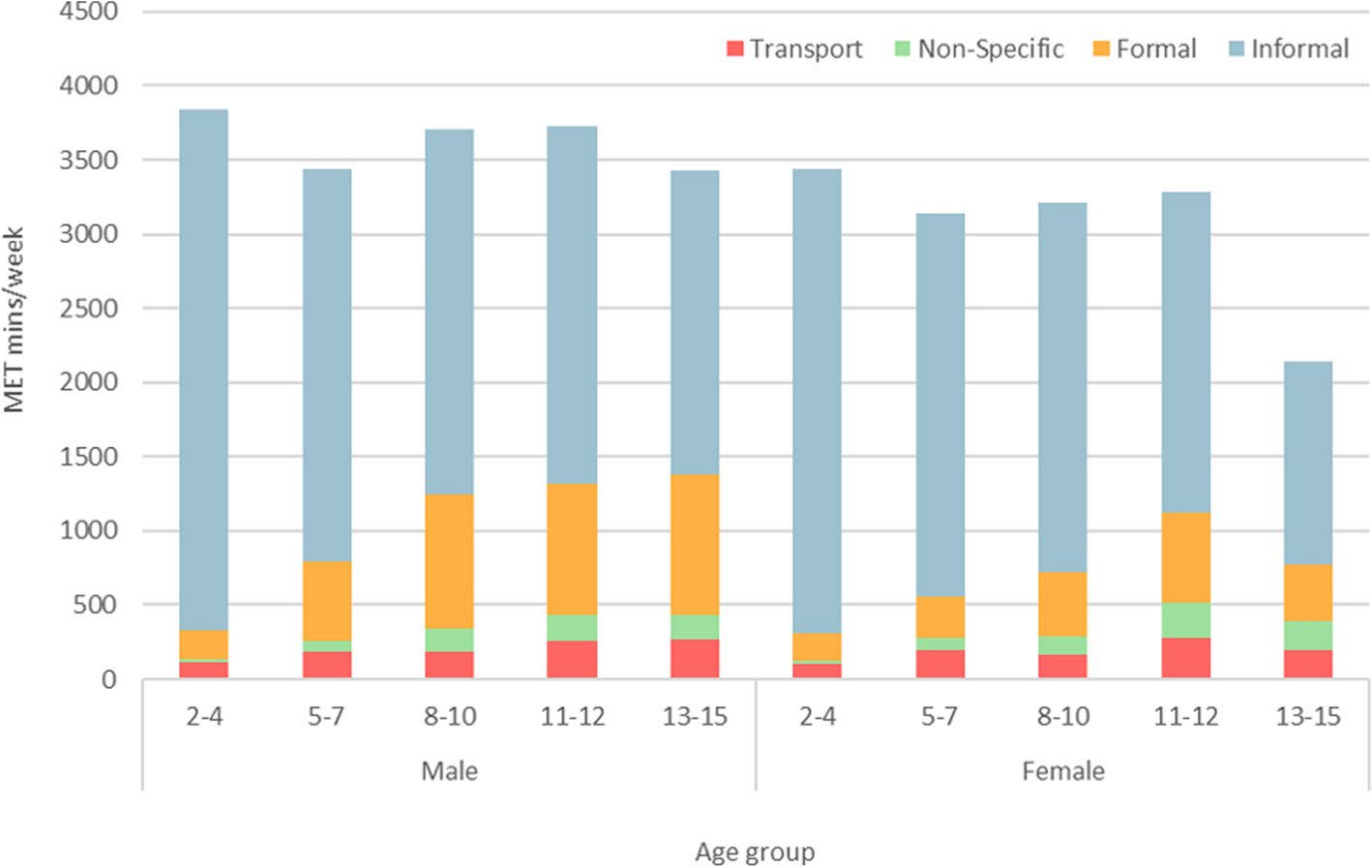
Relative domain-specific contributions towards mean MET mins/week from HSE 2012, by age and sex

Within the HSE 2012, age predicted outcomes on total MET minutes per week, and on all domain specific physical activity, controlling for sex. Overall, age was positively correlated with active travel, non-specific, and formal physical activity, but negatively with total and informal physical activity. Domain-specific contributions from each domain to total MET minutes per week are presented in Fig. 1. Further analyses were run stratifying for sex, finding age a significant predictor of all domain-specific outcomes. However, while age was a predictor of total MET minutes per week for girls (*F*_4_ = 6.42, *p* < 0.001) it was not for boys (*F*_4_ = 1.19, *p* = 0.312).

**Fig. 2:**
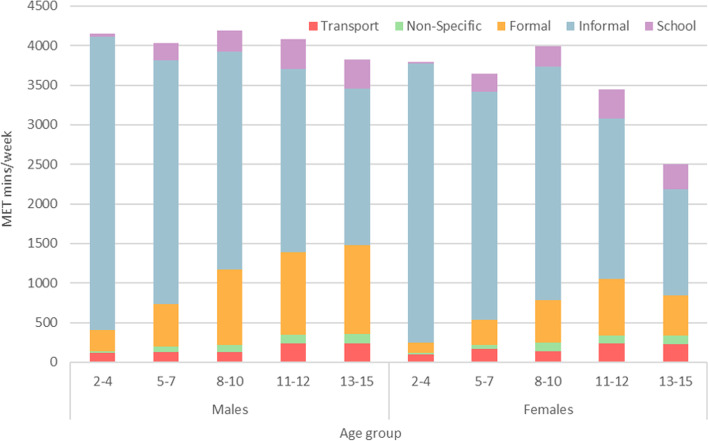
Relative domain-specific contributions towards mean MET mins/week from HSE 2015, by age and sex

For the HSE 2015 data, age again predicted outcomes on total MET minutes per week, both including and excluding school physical activity, and predicted all domains of physical activity. Age was once more positively correlated with totals for active travel, non-specific physical activity, formal activity, and for school-based activity, and was negatively correlated with informal activity and total MET minutes per week both including and excluding the contribution from school time. Domain-specific contribution to total MET minutes per week is presented in Fig. 2. Age remained a predictor for all domains when stratifying by sex; however, and while total activity differed significantly by age for girls (*F*_4_ = 17.48, *p* < 0.001), this was not the case for boys (*F*_4_ = 1.47, *p* = 0.208).

The HSE 2015 incorporated a measure of physical activity within curriculum time, the first time this had been included in any HSE iteration. The relative contributions of school activity are shown in Fig. 2, stratified by age and sex. There were significant differences in school physical activity levels for both sexes, with age predicting differences in school-based MET minutes per week for both boys (*F*_4_ = 106.54, *p* < 0.001) and girls (*F*_4_ = 101.30, *p* < 0.001).

### Effect by weight status

For the HSE 2012 data, weight status did not predict either total MET minutes per week (*F*_2,7_ = 0.85, *p* = 0.428) nor any domain-specific total, nor did any significant effects emerge when stratifying by sex. On the HSE 2015, weight status significantly predicted total MET minutes per week both including (*F*_2,7_ = 6.04, *p* = 0.002) and excluding (*F*_2,7_ = 6.10, *p* = 0.002) school activity, which persisted for girls (*F* = 4.20, *p* = 0.015), but not boys (*F*_2,6_ = 2.19, *p* = 0.112), when stratifying by sex. Increasing weight status also predicted reductions in informal activity (*F*_2,6_ = 4.82, *p* = 0.008). When stratifying by sex, girls (*F* = 5.37, *p* = 0.005), but not boys (*F*_2,6_ = 0.91, *p* = 0.402), retained a significant effect for informal activity, with no specific effect by gender on formal activity.

### Effect by deprivation

Within the HSE 2012, total MET minutes per week did not differ by QIMD for boys or girls. Neither were there significant effects for QIMD across domain-specific totals except for formal physical activity, which differed significantly (*F*_4,9_ = 3.98, *p* < 0.001), an effect which persisted when stratified by sex for girls (*F*_4,8_ = 4.78, *p* < 0.001), but not boys (*F*_4,8_ = 1.24, *p* = 0.292).

For HSE 2015, total MET minutes per week varied by QIMD category when including (*F*_4,9_ = 2.38, *p* = 0.049) or excluding (*F*_4,9_ = 2.55, *p* = 0.037) school activity, although neither effect persisted when stratifying for sex. Increasing QIMD was negatively associated non-specific, formal, and school activity levels, but positively associated with levels of informal activity. Stratification by sex revealed differences persisted on all domains of activity, except for active travel and school-based activity.

### Effect by ethnicity

Within the HSE 2012, participants from different identified ethnicities showed a significant variation on total MET minutes per week (*F*_4,9_ = 6.02, *p* < 0.001) when controlling for age and sex. At domain level, there were no large differences on active travel or non-specific activity levels but there were effects on formal (*F*_4,9_ = 5.83, *p* < 0.001) and informal (*F*_4,9_ = 4.54, *p* = 0.001) activity.

For the HSE 2015, different identified ethnicities showed a significant variation on total MET minutes per week both when including (*F*_4,9_ = 28.97, *p* < 0.001) and excluding (*F*_4,9_ = 28.12, *p* < 0.001) school activity, when controlling for age and sex. There were significant effects for all domain-specific totals, which persisted when stratified by sex except for boy’s active travel (*F*_4,8_ = 0.79, *p* = 0.533) and school activity (*F*_4,8_ = 0.59, *p* = 0.667).

### Stratified analysis by levels of physical activity

**Fig. 3:**
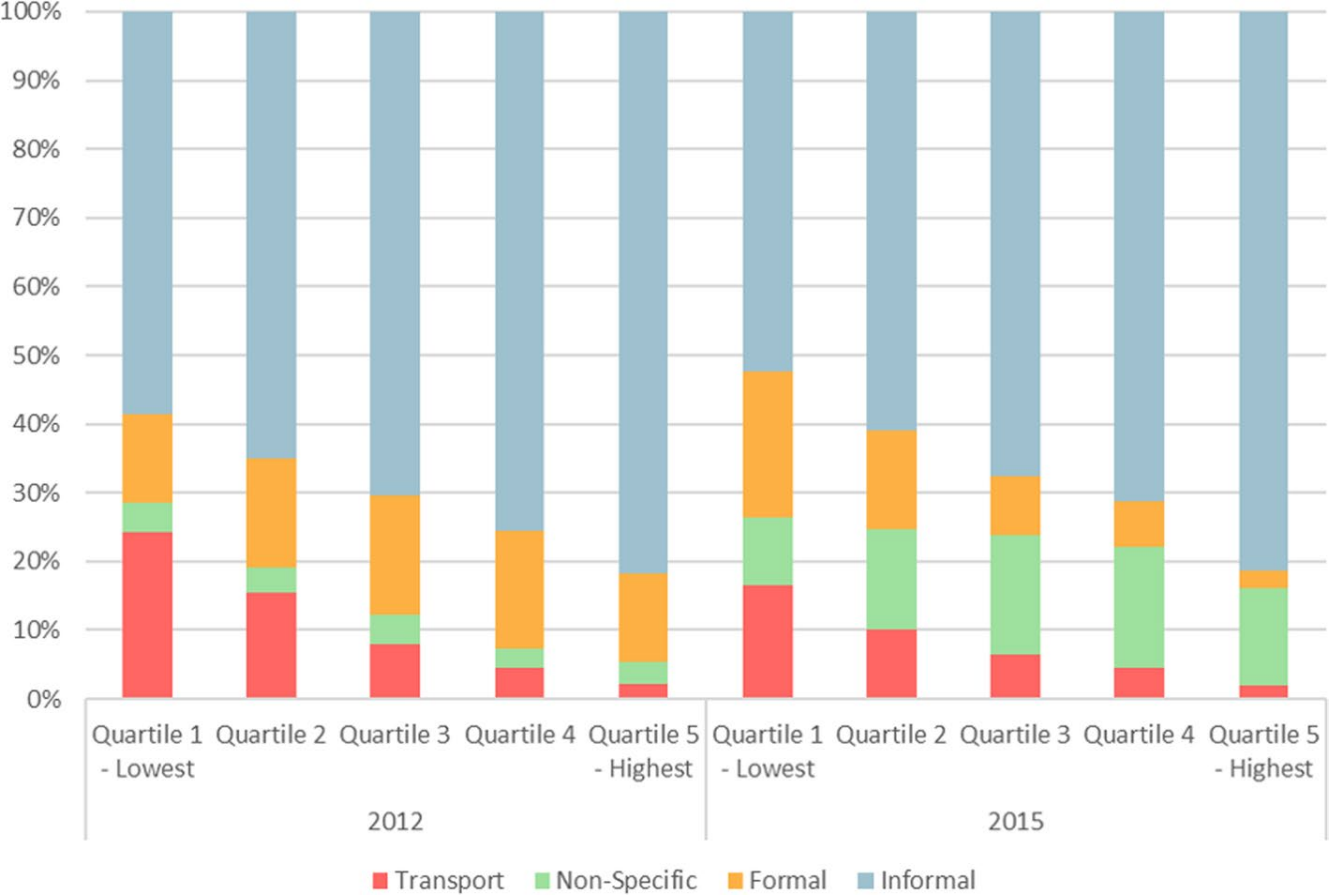
Relative contribution from domain-specific activity to total MET mins/week for HSE 2012 and HSE 2015, by activity strata

**Fig. 4:**
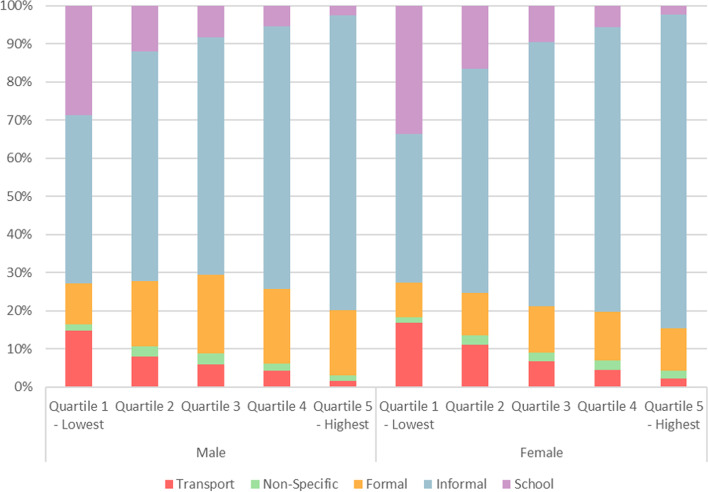
Relative contribution from domain-specific activity to total MET mins/week for HSE 2015, by activity strata

In addition to the above analyses of the whole survey sample, it is possible to stratify the responses according to levels of physical activity to provide a more nuanced description of the data and extending the work of Payne, Townsend [14]. Stratifying the HSE 2012 cohort by levels of physical activity reveals the relative contributions from each domain of physical activity to total MET minutes per week for activity-based quintiles of the population. For the HSE 2015 data, all domain-specific contributions showed significant regression effects by physical activity quintile when controlling for age and sex, effects which persisted when stratifying by sex. There were no statistically significant differences between survey years on comparable domains, although considerable variation appears when represented graphically. The percentage contributions across both HSE 2012 and HSE 2015 from comparable domains are presented in Fig. 3, with further data specifically including school activity from HSE 2015 presented in Fig. 4.

## Discussion

Analysis of both surveys revealed that boys engage in higher levels of physical activity than girls, both in terms of time spent in activity and in relative energy expenditure expressed in MET minutes per week, broadly matching the majority of findings from tracking studies [4, 5, 16, 28, 29]. Boys reported significantly larger absolute counts of MET minutes per week on the majority of domains of activity, with the exception of active travel. In terms of the relative contribution from specific domains, the most marked difference between sexes was participation in formal sport, where boys participate in a significantly higher amount of physical activity than girls throughout childhood. This difference was previously reported by Payne, Townsend [14] although their analysis was limited to data for the most active children; this more recent and more comprehensive analysis confirms this difference persists over time and across inactive as well active groups.

When stratified by age and sex, a consistent pattern of physical activity within the population appears across both 2012 and 2015. While active travel and non-specific activity remain comparatively stable, there is a steady decline in informal activity throughout childhood, and most dramatically in girls as they reach their mid-teens. In contrast, an increase in boy’s participation in formal activity appears to counteract this reduction in informal activity, with participation rates broadly consolidating by nine to ten years of age, but no such increase was apparent in girls. This finding clearly reinforces earlier work suggesting this declining pattern is evident in girls [30–32], and that there is a potential role for sports participation in addressing declines in activity through adolescence [33, 34].

There were few differences when stratifying physical activity by QIMD, in line with other research on this subject [16, 35]. Overall, a slight pattern of declining physical activity levels was observed as relative deprivation increased across all domains although mean levels of physical activity due to active travel, comprising walking or cycling to and from school, were largely robust to the influences of deprivation. Previous research (e.g. [36, 37]) indicates weight status is correlated with deprivation, although not necessarily with physical inactivity [38], confirmed by analysis of previous HSE iterations by Stamatakis, Wardle [39] and with significant post hoc correlations between weight status and QIMD in both the 2012 and 2015 iterations.

Perhaps surprisingly, although there was a mild trend of lower levels of physical activity with rising weight status, more evident in the larger 2015 sample, these differences were comparatively mild compared to what might be expected if weight status and physical activity levels were strongly associated. In some domains, and most particularly on the 2012 dataset, reported physical activity increased as weight status increased. This was in broad contrast to some (e.g. [40]), but not all (e.g. [38]), published research and potentially suggests physical activity does not directly moderate weight status in childhood.

There were notable differences in reported physical activity when responses were stratified by identified ethnicity. White children consistently reported higher levels of physical activity across all domains than other broad ethnic groups. In addition, there were significant increases in physical activity observed between 2012 and 2015 within the white child sample, while no increases were noted in other ethnic groups. Across majority-Caucasian nations there is considerable agreement that white children are more active than children from minority ethnic groups [41–44], although there are likely to be interactions with QIMD and weight status [18].

Between the two iterations of the HSE, there were few differences in the amount and nature of physical activity by QIMD, or by reported weight status. Comparison between survey years by sex revealed few statistically significant differences, suggesting a relatively stable pattern of physical activity by sex. A larger number of small but statistically significant differences were identified on the 2015 dataset, likely due to the larger sample size.

As can be expected, mean values of school physical activity remained largely consistent across both age and sex, in the same way that active travel was a small but consistent contributor to overall levels of physical activity. School physical activity, within the 2015 dataset, and active travel, across both datasets, appeared largely robust to change across any demographic variable. When stratifying the sample by levels of physical activity into quintiles the role of school activity and active travel played a far higher role in contributing to overall physical activity for the least active children. The major reductions were noted in formal and informal activity as people became less active.

The consideration of relative contribution of domain specific physical activity by physical activity quintile represents a unique contribution to the literature. Firstly, the contribution from non-volitional physical activity, specifically school-based activity and active travel, is increasingly important as overall physical activity levels reduce, with these domains contributing a vital source of physical activity for the least active children. Secondly, the important contribution of informal physical activity to total physical activity levels across all strata of physical activity is evident, with the majority of total physical activity being recruited form non-formal, largely volitional physical activity.

The above analysis provides a detailed exploration of the childhood physical activity data from the 2012 and 2015 iterations of the HSE, the last two iterations including a survey module on children’s physical activity. Overall, it appears that school curriculum-time activity, and active travel to and from school, provide a small but consistent contribution to weekly physical activity levels irrespective of age and sex, with this contribution becoming greater and more important for the least active children. Informal physical activity, largely consisting of play, but also including walking, cycling and (for girls) dancing, shows a pattern of steady decline through the childhood years. In boys, an increase in formal physical activity (i.e. sport) participation to some extent compensates for the decline in informal physical activity, but the near absence of a corresponding compensation results in reduced physical activity in older girls.

While a comprehensive assessment of childhood physical activity patterns in England is an important addition to the literature, there are limitations within the current paper. Reliance on self-report data contains inherent challenges, most specifically that responses are subject to inaccurate recall and social desirability bias, with overestimation of physical activity compared to objective measures [45]. Measurement of childhood physical activity is especially problematic due to considerable volatility, typically underestimating physical activity in young children but overestimating for older children [46]. Caution is required when interpreting the school-based physical activity findings, as estimates were derived from a single item question. Additionally, it is important to caution that the analysis of two subsequent iterations of the HSE comprise two cross-sectional analyses of considerably different sizes, and identified differences should not be interpreted as trend changes in physical activity. Lastly, the above analyses involve numerous statistical tests, and consequent risk of committing a familywise error; as such it is recommended that results falling below highly significant (*p* < 0.001) are viewed with caution.

In terms of policy recommendations, the relative contribution of active travel and school curriculum physical activity appear to be more meaningful in the less active groups, potentially providing important and equitable sources of physical activity to the least active children. We recommend these domains are protected and supported by potentially making system and/or environmental changes to facilitate sustained participation in these activities throughout childhood and beyond. Girls’ physical activity expenditure declines in the teenage years, most particularly due to a reduction in informal activity. In boys, a similar decline is mitigated by increases in formal sport, a mitigation largely absent in girls. As such, we recommend continued investment in increasing female participation in formal, but also informal, physical activity in adolescence. Finally, we recommend a more nuanced needs analysis is conducted when seeking to intervene and facilitate improvement in areas with varied ethnic minorities, rather than taking a more generic approach, as physical activity profiles vary between ethnic groups.

## Conclusions

Within the English population, boys typically report higher levels of physical activity in all behavioural domains at all ages. Furthermore, while both boys and girls report declining levels of physical activity through childhood, largely due to a steep decline in informal activity with age, only boys largely arrest this decline with increasing engagement in formal activities. Active travel and school physical activity contribute a consistent amount of activity throughout childhood and across all stratification variables and as a result constitute important sources of energy expenditure among the least active children.

## Data Availability

Datasets from the Health Survey for England are available from the NHS digital: https://digital.nhs.uk/data-and-information/publications/statistical/health-survey-for-england

## Abbreviations

HSE: Health survey for England
MET: Metabolic equivalent
PASBAQ: Physical activity and sedentary behaviour assessment questionnaire
UCL: University college london
NHS: National health service
QIMD: Quintile of index of multiple deprivation
